# In Vitro Comparative Study on Oppositely Charged Donepezil-Loaded Intranasal Liposomes

**DOI:** 10.3390/pharmaceutics17101250

**Published:** 2025-09-24

**Authors:** Elika Valehi, Gábor Katona, Dorina Gabriella Dobó, Ildikó Csóka

**Affiliations:** Faculty of Pharmacy, University of Szeged, Institute of Pharmaceutical Technology and Regulatory Affairs, Eötvös Str. 6, H-6720 Szeged, Hungary; valehi.elika@szte.hu (E.V.); csoka.ildiko@szte.hu (I.C.)

**Keywords:** liposome, nasal administration, dicethyl phosphate, stearylamine

## Abstract

**Background/Objectives**: Intranasal delivery is a promising approach for targeting the central nervous system (CNS); however, most of the drugs show poor permeability through the nasal mucosa. Nanocarriers such as liposomes can improve nasal drug absorption; however, the surface charge of liposomes has a key role in the nasal mucosal uptake process. Therefore, the present study aimed to formulate and compare the intranasal applicability of oppositely charged liposomes loaded with donepezil hydrochloride (DPZ) as CNS-active model compound using two different charge inducers, the negatively charged dicethyl phosphate (DCP) and the positively charged stearylamine (SA). **Methods**: Liposomes were prepared with a fixed phosphatidylcholine (PC)/cholesterol (CH) 7:2 molar ratio, while the effect of DCP and SA was studied in a 0.5:2 molar ratio. The most important properties for intranasal administration were studied, e.g., colloidal parameters, drug release and permeability behavior, and mucoadhesion. **Results**: It has been revealed that the reduction in liposome vesicle size is directly proportional to the amount of DCP, while it is inversely proportional to the amount of SA. This was also supported by the drug release studies—the lower vesicle size resulted in faster drug release. Both charge inducers increased the drug encapsulation efficiency (~60–80%) through tighter packing or increased spacing of the lipid bilayer structure. DCP also improved the in vitro nasal permeability compared to the initial DPZ solution. The positively charged SA showed more remarkable mucoadhesive properties than DCP. **Conclusions**: We can conclude that both charge inducers can be useful for improving nasal absorption of liposomal carriers, DCP in higher (PC:CH:DCP 7:2:2), while SA in lower concentrations (PC:CH:SA 7:2:0.5).

## 1. Introduction

Donepezil hydrochloride (DPZ) is a specific and reversible cholinesterase inhibitor (ChEI), which can be considered as first-line treatment of Alzheimer’s disease [1]. The conventional administration route of DPZ is the oral route, as its relative oral bioavailability is nearly 100%. However, various side effects, such as nausea and diarrhea, systemic dilution, substantial hepatic first-pass metabolism, and high plasma protein binding, were recognized after oral administration [2]. Moreover, the adequate therapeutic effect of DPZ is limited due to the blood–brain barrier (BBB), which restricts the brain penetration of a large number of therapeutic drugs [3].

Intranasal delivery is a promising alternative for targeting the central nervous system (CNS) [4,5,6]. Drugs can be absorbed through the highly vascularized nasal mucosa directly into the brain, bypassing the BBB, which offers the opportunity of effective treatment of several neurodegenerative diseases [7,8]. Numerous studies revealed that the intranasal administration of DPZ, particularly when formulated in nanoparticles or gels, has shown enhanced nasal permeability and improved bioavailability compared to oral or intravenous routes [9,10,11].

Liposomes are suitable nanocarriers for nasal drug delivery to enhance the absorption of drugs and therefore to improve their bioavailability when administered through the nasal route. As vesicular nanocarriers, they can protect the drug from enzymatic degradation and prolong the residence time on the nasal mucosa. This is particularly important for poorly permeable drugs, such as peptides, proteins, or vaccines [12,13]. However, the surface charge of liposomes can significantly impact nasal absorption. In general, positively charged liposomes exhibit improved nasal absorption compared to negatively charged or neutral liposomes due to the enhanced electrostatic interaction with the negatively charged nasal mucosa [14]. This interaction can increase the residence time of the drug on the nasal mucosa due to the increased resistance to mucociliary clearance and promote increased drug penetration through the nasal epithelium. Intranasal delivery of DPZ with different nano drug delivery systems has been explored in previous studies; for improving the colloidal or biopharmaceutical properties, different charge inducers were also investigated (Table 1). However, there is a lack of studies investigating the negatively charged dicethyl phosphate (DCP) or the positively charged stearylamine (SA) as frequently applied charge inducers for liposomal formulations.

The present study aimed to formulate and compare the intranasal applicability of oppositely charged liposomes loaded with DPZ as a CNS-active model compound using two different charge inducers, the negatively charged DCP and the positively charged SA.

## 2. Materials and Methods

### 2.1. Chemicals

Donepezil hydrochloride (DPZ, ≥98%), cholesterol (CH, ≥99%), phosphatidylcholine (PC, ≥99%) from soybean, stearylamine (SA, ≥99%), dicethyl phosphate (DCP), and porcine mucin (Type III) were purchased from Merck Hungary Ltd. (Budapest, Hungary). Phosphate-buffered saline (PBS, pH 5.6, containing 8.0 g/L NaCl, 0.2 g/L KCl, 1.44 g/L Na_2_HPO_4_·2H_2_O, and 0.12 g/L KH_2_PO_4_) and simulated nasal electrolyte solution (SNES, pH 5.6 containing 8.77 g/L NaCl, 2.98 g/L KCl, 0.59 g/L anhydrous CaCl_2_) were freshly prepared prior to the biopharmaceutical investigations. Ethanol (96% *v*/*v*) was obtained from Molar Chemicals Ltd. (Budapest, Hungary). All other reagents were purchased from Merck Hungary Ltd. (Budapest, Hungary), if not indicated otherwise.

### 2.2. Preparation of Liposomes

**Figure 1:**
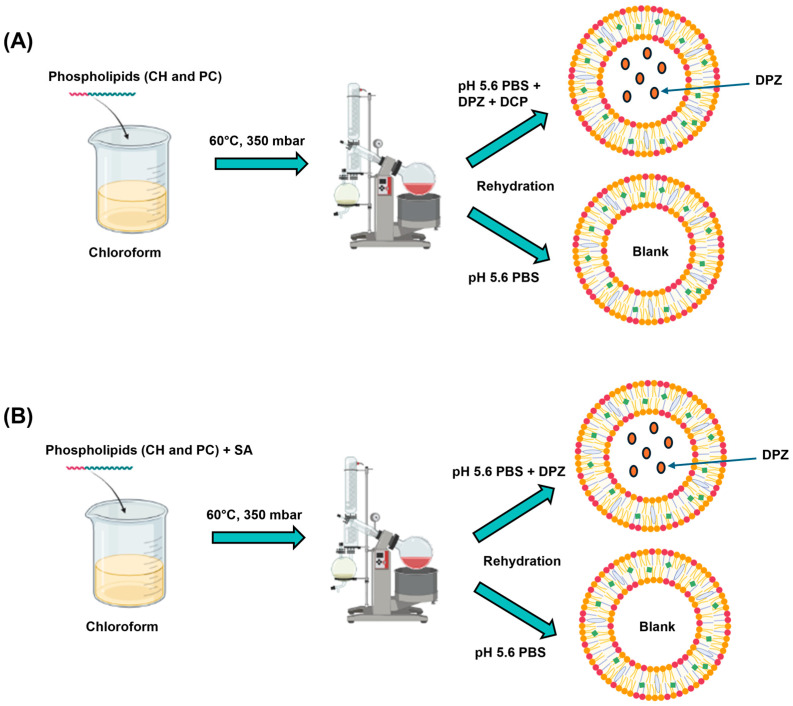
The main preparation steps of liposomal formulations with different charge inducers: DCP (**A**) and SA (**B**).

Blank and DPZ-loaded liposomes were created using a thin-film hydration technique with a uniform PC:CH molar ratio (2:7), which was selected based on preliminary experiments. Briefly, PC and CH (5 mg together) were dissolved in 2 mL of chloroform in round-bottom flasks. In the case of cationic liposomes, SA was also dissolved along with the lipids in different molar ratios (0.5, 1, 1.5, and 2), as it is insoluble in water. After that, the organic solvent (chloroform) was evaporated using a rotary vacuum evaporator (Büchi Rotavapor^®^ R-215, BÜCHI Labortechnik AG, Flawil, Switzerland) at 60 °C and 350 mbar pressure with 25 rpm, forming a lipid film on the wall of the round-bottom flask. For the hydration of the lipid, 5 mL of pH 5.6 PBS (for blank liposomes) or 5 mL of DPZ solution (1 mg/mL in pH 5.6 PBS) was added, and ultrasonication (Elmasonic S 30 H, Elma Schmidbauer GmbH, Singen, Germany) was applied at 60 °C for 30 min to form liposomes. In the case of anionic liposomes, DCP was dissolved in the PBS in different molar ratios (0.5, 1, 1.5, and 2) prior to hydration, as it is a water-soluble excipient. The main preparation steps are shown in Figure 1, whereas the composition of liposomal carriers is presented in Table 2. Thereafter, vacuum filtration (Rocker 400 oil-free vacuum pump, Rocker Scientific Co., Ltd. New Taipei City, Taiwan) was performed in two steps. First, the liposomal solution was filtered using a 0.45 µm nylon membrane disk filter (47 mm, Labsystem Kft., Budapest, Hungary), then the solution was further filtered through a 0.22 µm membrane filter (Ultipor^®^ N66 nylon 6.6 membrane disk filter 47 mm, Pall Corporation, New York, NY, USA). The obtained liposomes were freeze-dried at reduced pressure (0.015 mbar) at −40 °C for 12 h (primary drying) and at 25 °C for an additional 4 h (secondary drying) using a SanVac CoolSafe freeze-dryer (LaboGeneTM, Lillerød, Denmark) to ensure long-term storage stability.

### 2.3. Determination of Average Hydrodynamic Diameter, Polydispersity Index, and Surface Charge

Dynamic light scattering was used to determine the colloidal parameters of liposomal formulations (average hydrodynamic diameter (Z-average), polydispersity index (PdI), and zeta potential). Samples were transferred in folded capillary cells and measured with a Malvern nano ZS instrument (Malvern Instruments, Worcestershire, UK) at room temperature (25 °C) with the refractive index of 1.445. Three parallel measurements were performed; the data were presented as means ± SD.

### 2.4. Encapsulation Efficiency Determination

To determine the encapsulation efficiency (EE), liposomal formulations were centrifuged for 15 min at 4 °C and 14,000 rpm (Hermle Z323 laboratory centrifuge, Hermle AG, Gossheim, Germany). Then, the supernatant was collected, and free DPZ content was measured by HPLC. Three parallel measurements were performed; data were presented as means ± SD. The EE of DPZ-loaded liposomes was calculated using the following equation [20]:

(1) $$Encapsulation\;efficiency(\%)=\frac{W_{total}-W_{free}}{W_{total}}\times 100$$

where *W_total_* was the total weight of DPZ in liposomal formulations (mg) and *W_free_* was the weight of free DPZ in liposomal formulation (mg).

### 2.5. In Vitro Drug Release Studies

The two-stage biorelevant drug release study was performed at nasal conditions using the modified paddle method (Hanson SR8 Plus, Teledyne Hanson Research, Chatsworth, CA, USA) at 32 °C at 100 rpm [21]. The freeze-dried formulations were redispersed in 1 mL purified water (with 1 mg/mL DPZ nominal concentration). Then, 1 mL of liposomal formulations and reference DPZ solution were transferred into pre-treated dialysis bags (Spectra/Por^®^ Dialysis Membrane) with an MWCO value of 12–14 kDa (Spectrum Laboratories Inc., Rancho Dominguez, CA, USA) and sealed at both ends. As a dissolution medium, first 100 mL of SNES was used for 15 min (to mimic the pH and electrolyte composition of nasal fluid), then loaded dialysis bags were transferred into 100 mL of pH 7.4 PBS (to represent central nervous system conditions) for an additional 225 min. Aliquots were taken at predetermined time intervals and replaced with fresh medium. The actual DPZ content of aliquots was determined by HPLC. Each measurement was carried out in triplicate; the data were shown as mean ± SD.

### 2.6. Droplet Size Distribution Study

The droplet size distribution of the liposomal formulations was determined with laser diffraction using a Malvern Spraytec^®^ instrument (Malvern Instruments Ltd., Malvern, UK) equipped with a 300 mm lens. Droplet size analysis was performed in the range of 0.1–900 μm (Dv50: 0.5–600 μm). The nasal spray container was positioned horizontally to the receiving lens to reach the laser beam precisely in the center of the expansion of the spray cone. Three parallel measurements were performed with each liposomal formulation at room temperature. Measurement data were evaluated with the Spraytec^®^ software v4.00 (Malvern Panalytical Ltd., Malvern, UK), the volume diameter 10% (Dv10), 50% (Dv50), and 90% (Dv90) of the cumulative volume distribution was determined. The results were presented as means ± SD. The Span value was calculated using the following equation [22]:

(2) $$Span=\frac{\left(Dv90-Dv10\right)}{Dv50}$$

### 2.7. In Vitro Drug Diffusion Studies

The nasal diffusion study of liposomal formulations was performed at 32 °C in a modified Side-Bi-Side^®^-type horizontal diffusion cell system [23]. As an artificial nasal membrane, an isopropyl myristate-impregnated cellulose membrane (regenerated cellulose, Whatman™, 0.45 µm, 25 mm, GE Healthcare Sciences, Chalfont St. Giles, UK) was used between the two compartments. The freeze-dried formulations were redispersed in 1 mL of purified water (with 1 mg/mL of DPZ nominal concentration) and added to the donor phase, which consisted of 8.0 mL of SNES, while the acceptor phase contained 9.0 mL of pH 7.4 PBS. As a reference, 1 mL of DPZ solution was used. Aliquots from the acceptor phase (150 µL) were taken at predetermined time intervals for 60 min and replaced with fresh medium. Actual DPZ content in the aliquots was determined using HPLC.

The steady-state flux (*J_ss_*) was calculated, as seen in the following equation:

(3) $$J_{ss}=\frac{m_{t}}{A\;\times \;t}$$

where *Jss* means the steady-state flux (µg/cm^2^/h), *m_t_* the amount of drug permeated through the membrane, *A* the surface of the membrane insert (0.785 cm^2^), whereas *t* the duration of the measurement (1 h).

### 2.8. HPLC Method

The quantification of DPZ was performed via HPLC (Agilent 1260, Agilent Technologies, Santa Clara, CA, USA). As a stationary phase, a Kinetex^®^ EVO C18 column (150 mm × 4.6 mm, 5 µm, Phenomenex, Torrance, CA, USA) was used. As a mobile phase, 0.02 M PBS (pH 2.7) with 1% *v*/*v* triethylamine (A) and methanol (B) was used in a 50:50 ratio. A total of 10 μL samples were analyzed, the isocratic separation was performed at 25 °C with 1 mL/min eluent flow [24]. Chromatograms were analyzed at 268 nm using a UV-VIS diode array detector using ChemStation B.04.03 Software (Santa Clara, CA, USA).

### 2.9. Mucoadhesion Studies

The mucoadhesive property of liposomal preparations was investigated with the turbidimetric method by studying the interaction with mucin. Equal volumes of reconstituted liposomal formulations and mucin solution (0.5% *w*/*v* in SNES) were mixed, transferred into vials, and incubated at 37 °C for 2 h. At predetermined time intervals (15, 30, 60, and 120 min), the samples were centrifuged at 14,000 rpm for 10 min at 4 °C (Hermle Z323 laboratory centrifuge, Hermle AG, Gossheim, Germany), and the free mucin content was quantified at 255 nm using a Jasco V-730 UV spectrophotometer (ABL&E JASCO Ltd., Budapest, Hungary). The mucin binding efficiency (MBE) of the liposomal formulations was calculated using the following equation [25,26,27]:

(4) $$MBE\left(\%\right)=\frac{Total\;mucin\;used-Free\;mucin}{Total\;mucin\;used}\times 100$$

### 2.10. Statistical Analysis

Statistical analysis of colloidal parameters, drug release, and drug permeability studies was performed with one-way ANOVA with post hoc test (Tukey’s multiple comparisons test, α = 0.05). Statistical significance of results was considered when *p* < 0.05.

## 3. Results and Discussion

### 3.1. Selection of Liposomal Composition

Selection of liposomal composition was based on safety considerations. Regarding mucosal toxicity, DCP does not appear to be known as a hazardous excipient. A Cosmetic Ingredient Review (CIR) Expert Panel concluded that DCP is safe for use in cosmetics at appropriate concentrations, including nasal applications, when formulated to be a non-irritant. In the case of SA toxicity, issues can arise at certain concentrations. There is a lack of experimental data for nasal irritation or toxicity; however, Senior et al. investigated the interaction of cationic liposomes on erythrocytes [28]. Based on their findings, SA above 0.5 µmol/mL can cause hemolysis, which can also be significant in the case of nasal absorption. Therefore, in the present study, the maximum concentration of SA applied in the formulations was set to 0.15 µmol/mL to avoid toxicological issues. The selected molar ratio of DCP has been adjusted to the molar of SA for easier comparison.

### 3.2. Characterization of Colloidal Parameters

For adequate nasal absorption, colloidal parameters are crucial. Generally, the optimal Z-average can be considered in the range of 100–200 nm, the PdI of 0–0.3, and absolute value of zeta potential higher than 15 mV, respectively [29]. Charge inducers, such as DCP or SA, can affect colloidal parameters depending on their concentration. DCP, as a negative charge inducer, can improve the interaction of liposomes with the nasal mucosal surface, achieving improved drug delivery, as well as inhibit the aggregation of liposomal vesicles due to electrostatic repulsion. Studies have revealed that increasing the concentration of DCP can reduce vesicle size and even zeta potential [30,31]. However, DCP concentration should be carefully optimized, as its application in too high concentrations can lead to excessive aggregation (high PdI) and instability.

**Figure 2:**
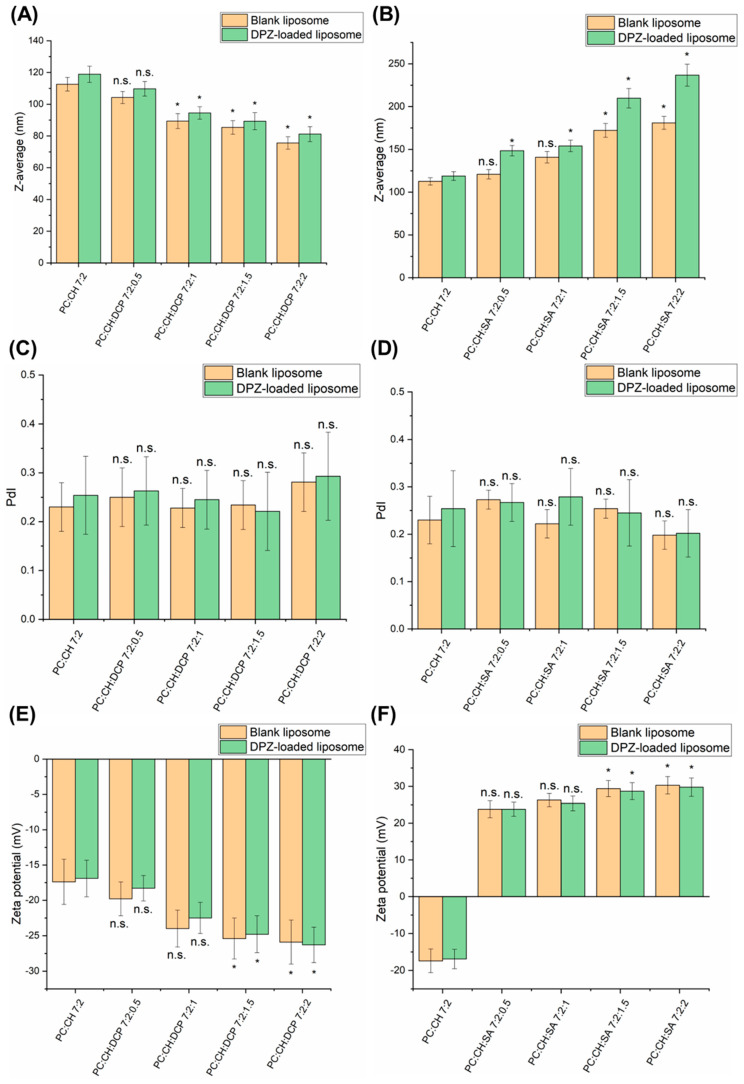
Colloidal parameters (Z-average, PdI, and zeta potential) of DPZ-loaded liposomal formulations compared with blank liposomes: (**A**) Z-average of DCP-liposomes; (**B**) Z-average of SA-liposomes; (**C**) PdI of DCP-liposomes; (**D**) PdI of SA-liposomes; (**E**) zeta potential of DCP-liposomes; (**F**) zeta potential of SA-liposomes; (n.s.) means marks non-significant, whereas asterisks indicate significant change (* *p* < 0.05). Data were presented as means ± SD (n = 3).

Our experiments revealed similar results; while increasing the concentration of DCP, the Z-average of liposomes decreased even lower than 100 nm (PC:CH:DCP 7:2:1, PC:CH:DCP 7:2:1.5 and PC:CH:DCP 7:2:2), as presented in Figure 2A. DPZ-loaded liposomes showed a slightly higher Z-average in comparison to corresponding blank liposomes, which can probably be explained by the higher encapsulation efficiency (EE) (Table 3). The EE results revealed both charge inducers can help to retain the drug within the liposomes due to electrostatic interactions. DCP, as an anionic lipid, results in tighter packing of the bilayer membrane due to double hydrocarbon chains, which contribute to reduced leakage, therefore increased EE. The effect of SA on drug encapsulation can be explained with different mechanisms, e.g., by modifying the surface charge of liposomes, creating a positive charge to attract and bind negatively charged drugs through electrostatic interactions, or forming ion pairs with acidic drugs. In the case of DPZ-liposomes, the most likely mechanism is an interaction between lipid components and SA, which can increase the spacing between lipid bilayers, enhancing EE of hydrophilic drugs as DPZ [32,33]. The formulations PC:CH:DCP 7:2:2 and PC:CH:SA 7:2:2 showed the highest EE values, indicating that the stabilizing effect (leakage prevention) on the liposomal bilayer increased proportionally with the concentration of both charge inducers. There was no trend observed in the PdI values; however, at the highest DCP concentration tested (PC:CH:DCP 7:2:2), the PdI value was still above 0.3, indicating a destabilizing effect (Figure 2C). In contrast, the increase in zeta potential absolute values was proportional to DCP concentration (Figure 2E).

SA is a cationic lipid, which has a positive charge at physiological pH due to containing an ionizable nitrogen atom in the structure [34]. SA incorporates asymmetrically in the lipid bilayer of liposomes oriented mainly to the outer surface of the liposomes [35]. Studies on SA-liposomes showed increased stability of the lipid bilayer, reduced leakage of encapsulated materials, and a controlled release profile. However, it can interact with certain enzymes, causing a cytotoxic effect, which limits clinical use [36]. Studies have reported that SA can increase the size of liposomes due to electrostatic repulsion between the positively charged SA molecules and the liposome surface. However, it can reduce PdI due to the stabilization of the liposome membrane, therefore preventing aggregation [37,38].

Our experiment with SA reveals similar results in Figure 2B: the Z-average proportionally increased with the concentration of SA; however, still the highest SA concentration tested (PC:CH:SA 7:2:2) fit the acceptance criteria of nasal administration (for Z-average 100–200 nm, for PdI 0.0–0.5 and for zeta potential <−15 or >+15 mV [29]). Similarly to DCP, in the case of SA, the DPZ-loaded liposomes also showed a slightly higher Z-average in comparison to corresponding blank liposomes, which can be probably explained by the higher EE (Table 3). Also, in the case of PdI and zeta potential, the stabilizing effect (narrowing PdI and increasing zeta potential) was observed by increasing the concentration of SA (Figure 2D,F).

**Figure 3:**
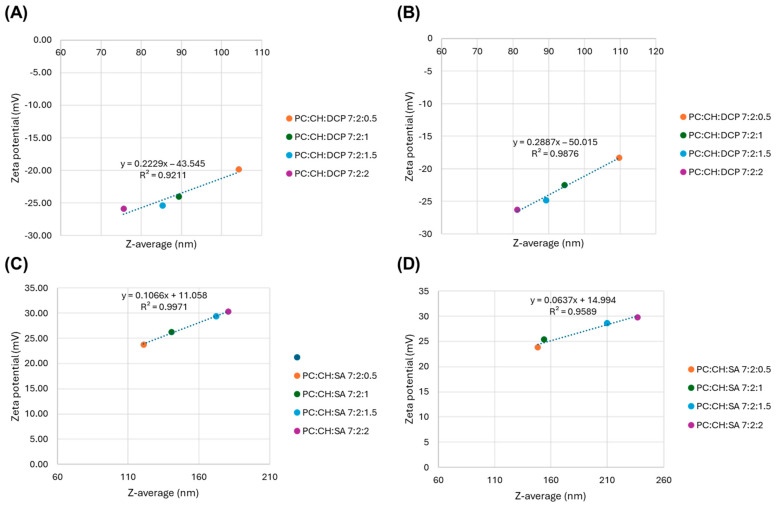
Correlation plot between Z-average and zeta potential of blank DCP-liposomes (**A**), DPZ-loaded DCP-liposomes (**B**), blank SA-liposomes (**C**) and DPZ-loaded SA-liposomes (**D**).

The correlation between colloidal parameters was also investigated. A good correlation was observed between the Z-average and zeta potential values, as presented in Figure 3, which supports the influence of the concentration of applied charge inducers on the colloidal parameters.

### 3.3. Droplet Size Distribution

Droplet size distribution is an important parameter of in vitro bioavailability of liquid nasal formulations. A typical droplet size (Dv50) ranges between 20 and 200 µm [39]. Based on the literature, droplets with Dv50 lower than 10 µm could reach the lungs through the nasopharynx during inhalation [40] or could be deposited in the anterior region of the nasal cavity and may be eliminated fast through the mucociliary clearance [41]. The results revealed that all tested liposomal formulations are suitable for adequate nasal deposition based on the Dv50 values (Table 4). Droplet size spread (Span) values are used to evaluate the width of droplet size distribution. All prepared formulations showed low Span values (0.48–0.86), which correlate with a narrow droplet size range. Increasing the concentration of both charge inducers (DCP and SA) resulted in a decrease in droplet size, which can be claimed with the reduction in surface tension.

### 3.4. Drug Release of Liposomal Formulations

**Figure 4:**
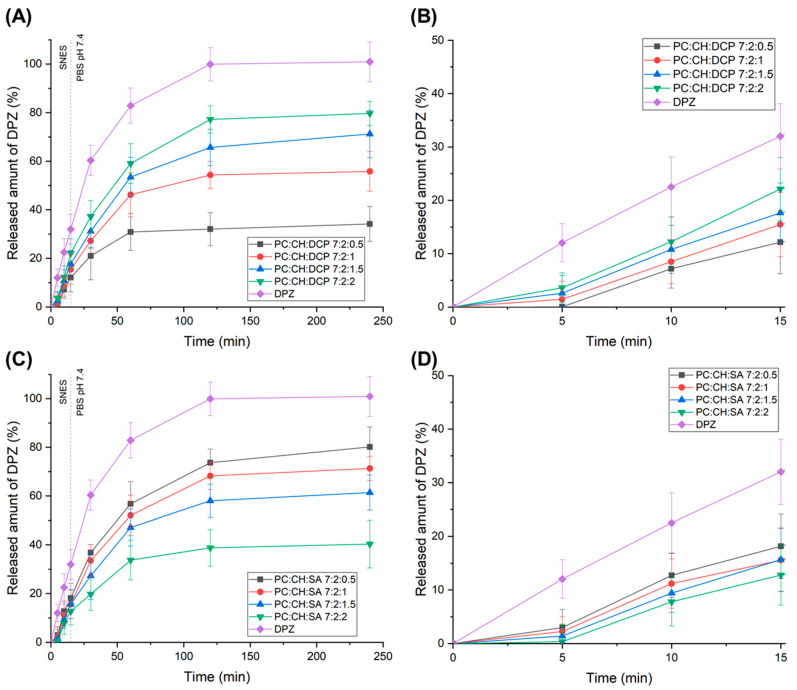
In vitro drug release profiles of DCP- (**A**) and SA-liposomes (**C**) magnified to reflect nasal cavity conditions during the first 15 min (**B**,**D**).

Drug release studies were conducted in a two-stage biorelevant release test under nasal conditions (Figure 4). First, the drug release of DPZ-loaded liposomal formulations and reference DPZ solution was investigated in SNES for 15 min, mimicking the nasal mucosal environment. Under physiological conditions, the residence time of the administered formulations on the nasal mucosa does not exceed 15 min due to mucociliary clearance. Then, the dialysis bags (containing the formulations) were transferred into PBS 7.4 medium (central nervous system conditions) for an additional 225 min. Drug release studies revealed that in the case of both charge inducers, the drug release rate of liposomal formulations was lower than 20% within 15 min in SNES, which indicates a minimal free fraction of active substances in the nasal cavity. The higher part of the drug (~80%) can absorbed along with the liposomal carrier. In PBS 7.4, controlled release of liposomal formulations was observed, which indicates a prolonged therapeutic effect. Moreover, the dissolution extent was in accordance with the Z-average results—lower vesicle size resulted in faster drug release. In the case of DCP-liposomes, PC:CH:DCP 7:2:2 showed the fastest drug release, while in the case of SA-liposomes, the PC:CH:SA 7:2:0.5 was the most promising. These formulations indicated a significantly higher (*p* < 0.05) drug release rate after 120 min in comparison with the corresponding liposomal formulation containing the charge inducer in lower concentrations. Fang et al. described how DCP can increase the permeability of the lipid bilayer by creating and stabilizing pores or disordered regions, leading to increased drug release [42]. However, when increasing the SA concentration, the drug release was decreased, which can be claimed with the positive charge of both SA and DPZ. SA reduces the permeability of the cationic drugs like DPZ to lipid bilayers because of the electrostatic repulsion, therefore improving EE but hindering drug release [43]. Srinath et al. also described how the inclusion of SA in the liposomes reduced the release of the drug due to electrostatic interaction [44]. The most relevant drug release kinetic models (zero order, first order, Higuchi, Korsmeyer–Peppas, and Hixson–Crowell) were fitted, and kinetic parameters were calculated (Table S1). PC:CH:SA 7:2:0.5 and DPZ followed first-order kinetics, which indicates that the applied low molar ratio of SA had no influence on drug release. All other liposomal formulations followed Higuchi kinetics, suggesting that drug transport out of the liposomes was driven mainly by a diffusion-controlled mechanism. This finding can explain that the drug liberation from liposomes turns into a sustained release after 120 min. Similar results were obtained with DPZ-loaded liposomes by Vasileva et al. [45].

### 3.5. Drug Diffusion Test

**Figure 5:**
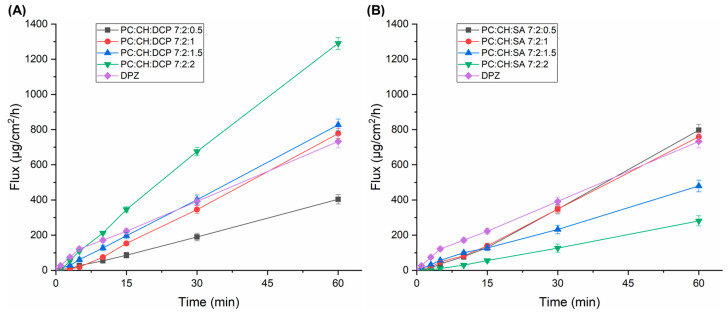
In vitro drug permeability profiles of DCP- (**A**) and SA-liposomes (**B**).

The modified Side-Bi-Side^®^-type horizontal diffusion cell was used for investigation of the in vitro nasal drug permeability through an artificial nasal membrane. The diffusion of DPZ-loaded liposomal formulations was compared to DPZ solution. Figure 5 shows the obtained cumulative permeability profiles, while Table 5 represents the flux values at t = 1 h.

In the case of DCP-liposomes, PC:CH:DCP 7:2:1 and PC:CH:DCP 7:2:1.5 showed similar permeability to the reference DPZ solution; however, in the case of PC:CH:DCP 7:2:2, the permeability was significantly higher (*p* < 0.05), which might be explained by the high concentration of DCP that can alter the structure of the lipid bilayer (Figure 5A). This alteration can lead to increased fluidity and a less ordered structure, effectively creating different pathways for lipophilic molecules such as DPZ (log *P* of 4.27) [46]. PC:CH:DCP 7:2:1 showed significantly lower (*p* < 0.05) flux value after 60 min, which supports the idea that DCP has a crucial effect in the permeability enhancement. In the case of SA-liposomes, PC:CH:SA 7:2:1.5 and PC:CH:SA 7:2:2 showed significantly lower (*p* < 0.05) flux values after 60 min in comparison to liposomes with lower SA concentration and DPZ solution, which can be claimed with the electrostatic repulsion between positively charged DPZ and SA (Figure 5B).

### 3.6. Mucoadhesive Properties

**Figure 6:**
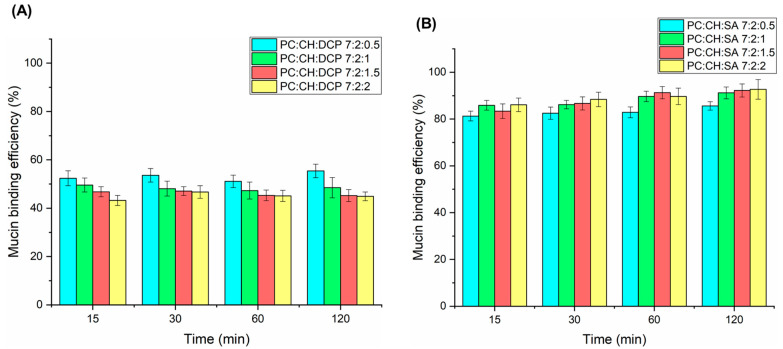
In vitro mucoadhesion study results of DCP- (**A**) and SA-liposomes (**B**).

Mucoadhesion is a crucial property of intranasal nanocarriers for improving residence time on the nasal mucosa, which ensures adequate drug absorption. Simple PC:CH liposomes show weak mucoadhesion; however, their surface modification with charged excipients or polymers can improve this property. The turbidimetric method revealed moderate mucin binding efficiency (MBE) for DCP-liposomes (44.9–55.4%); however, strong mucoadhesive properties were observed for all SA-liposomes within the 2 h investigation (85.6–92.7%), indicating attractive interaction between SA and mucin (Figure 6). No significant difference was observed between the concentration of charge inducers and their mucoadhesive properties. The mechanism of interaction of DCP with mucin can also be explained with the hydration of the hydrocarbon chain of DCP, which therefore interpenetrates between the mucin molecules. This phenomenon can also be likely for SA-liposomes, but here the mechanism is primarily driven by electrostatic interactions [47,48,49].

## 4. Conclusions

The present comparative study revealed that both charge inducers have a remarkable effect on the nasal applicability of liposomal carrier. The application of DCP in higher concentrations (PC:CH:DCP 7:2:1.5 and PC:CH:DCP 7:2:2) decreased the vesicles size, therefore accelerated the drug release and improved in vitro nasal permeability. In contrast, SA had a favorable effect in lower concentrations (PC:CH:SA 7:2:0.5 and PC:CH:SA 7:2:1). Moreover, due to the positive surface charge, SA showed beneficial mucoadhesive properties. Furthers cytotoxicity and in vivo studies would be advantageous to perform to further support the safety and efficacy of the two charge inducers.

## Figures and Tables

**Table 1:** Overview of different nano drug delivery systems and outcomes of DPZ nasal delivery systems.

Drug Delivery System	Formulation	Charge Inducer	Result/Outcome	Reference
Nanoemulsion	Labrasol Cetyl pyridinium chloride Glycerol	Cetyl pyridinium chloride	Z-average: 65 nm -PdI: 0.084 Zeta potential: −10.7 mV Release property: Fast release in PBS (pH 7.4), ACSF, simulated nasal fluid Cell viability: 76.3% (Neuro 2a)	[15]
Liposomes	Hydrogenated soy phosphatidyl cholin Cholesterol -Ammonium sulfate	Ammonium sulfate	Z-average: 103 nm PdI: 0.108 -EE: 93% Nasal mucosa permeation: 80.11% Mucoadhesive strength: 2320 dyne/cm^2^	[16]
Solid lipid nanoparticles (SLNs)	Compritol Capryol 90 Poloxamer 188 Chitosan	Chitosan	Z-average: 193 nm PdI: 0.298 Zeta potential: 38.9 mV EE: 89.85% Release property: sustained release (84.82%) up to 24 h Highly Mucoadhesive	[17]
Polymeric Lipid Nanoparticles	Soy lecithin Glutaraldehyde Chitosan Glacial acetic acid Gelatin	Chitosan	Z-average: 237 nm Zeta potential: positive Drug loading: 10.24% Release property: burst release of up to 99.99% for 5 days Cell viability: safe toward mouse fibroblast cells (L929) Highly Mucoadhesive	[18]
Nanosuspension	Chitosan Sodium tripolyphosphate Glacial acetic acid	Chitosan	Z-average: 177.8 nm PdI: 0.593 Zeta potential: 16.6 mV Release property: sustained, 90.82% (24 h) Mucodhesive force: 9.26 g	[19]

**Table 2:** Molar composition of liposomal carriers.

Formulation Code	Amount of Lipids		Amount of Charge Inducers	
	Molar Ratio of PC	Molar Ratio of CH	Molar Ratio of DCP	Molar Ratio of SA
PC:CH:DCP 7:2:0.5	7	2	0.5	0
PC:CH:DCP 7:2:1	7	2	1	0
PC:CH:DCP 7:2:1.5	7	2	1.5	0
PC:CH:DCP 7:2:2	7	2	2	0
PC:CH:SA 7:2:0.5	7	2	0	0.5
PC:CH:SA 7:2:1	7	2	0	1
PC:CH:SA 7:2:1.5	7	2	0	1.5
PC:CH:SA 7:2:2	7	2	0	2

**Table 3:** Encapsulation efficiency results of liposomal formulations.

Liposomal Formulation	EE (%)
PC:CH:DCP 7:2:0.5	58.4 ± 2.1
PC:CH:DCP 7:2:1	62.1 ± 2.7
PC:CH:DCP 7:2:1.5	69.6 ± 3.4
PC:CH:DCP 7:2:2	73.8 ± 1.8
PC:CH:SA 7:2:0.5	67.6 ± 3.9
PC:CH:SA 7:2:1	72.8 ± 4.1
PC:CH:SA 7:2:1.5	75.5 ± 2.2
PC:CH:SA 7:2:2	81.3 ± 2.7

**Table 4:** Droplet size distribution of liposomal formulations. Results are presented as means ± SD (n = 3).

Formulation	Dv10 (µm)	Dv50 (µm)	Dv90 (µm)	Span
PC:CH 7:2	131.2 ± 6.3	173.9 ± 11.4	228.2 ± 10.3	0.56 ± 0.21
PC:CH:DCP 7:2:0.5	125.4 ± 4.7	173.2 ± 8.8	203.1 ± 12.6	0.48 ± 0.13
PC:CH:DCP 7:2:1	113.2 ± 5.9	160.0 ± 6.3	258.9 ± 13.8	0.84 ± 0.17
PC:CH:DCP 7:2:1.5	110.4 ± 1.8	160.1 ± 4.1	232.7 ± 9.7	0.76 ± 0.09
PC:CH:DCP 7:2:2	101.4 ± 3.6	151.3 ± 8.7	198.4 ± 12.5	0.55 ± 0.23
PC:CH:SA 7:2:0.5	121.4 ± 7.4	184.3 ± 7.5	376.9 ± 24.6	0.72 ± 0.14
PC:CH:SA 7:2:1	118.4 ± 2.2	168.2 ± 5.3	260.2 ± 13.3	0.68 ± 0.18
PC:CH:SA 7:2:1.5	111.5 ± 6.8	171.8 ± 6.7	236.6 ± 15.1	0.86 ± 0.06
PC:CH:SA 7:2:2	81.1 ± 9.7	125.4 ± 4.1	186 ± 9.5	0.83 ± 0.02

**Table 5:** Flux values of liposomal formulations at the end of experiment (t = 1 h).

Formulation	Flux (µg/cm^2^/h) at t = 1 h
DPZ solution	732.81 ± 36.5
PC:CH:DCP 7:2:0.5	404.24 ± 26.7
PC:CH:DCP 7:2:1	777.33 ± 28.9
PC:CH:DCP 7:2:1.5	827.19 ± 33.4
PC:CH:DCP 7:2:2	1290.21 ± 33.6
PC:CH:SA 7:2:0.5	796.92 ± 33.1
PC:CH:SA 7:2:1	759.52 ± 34.2
PC:CH:SA 7:2:1.5	479.93 ± 27.2
PC:CH:SA 7:2:2	281.37 ± 26.3

## Data Availability

Data is available on request to the corresponding author.

## Supplementary Materials

The following supporting information can be downloaded at: https://www.mdpi.com/article/10.3390/pharmaceutics17101250/s1, Table S1: Drug release kinetic models and kinetic parameters of DPZ-loaded liposomes.

## Abbreviations

The following abbreviations are used in this manuscript:

DPZ	Donepezil hydrochloride
ChEIs	Cholinesterase inhibitors
BBB	Blood–brain barrier
CNS	Central nervous system
DCP	Dicethyl phosphate
SA	Stearylamine
CH	Cholesterol
PC	Phosphatidylcholine
SNES	Simulated nasal electrolyte solution
Z-average	Average hydrodynamic diameter
PdI	Polydispersity index
EE	Encapsulation efficiency
J_SS_	Steady-state flux
MBE	Mucin binding efficiency
