# Supplementary material for: In Vitro Comparative Study on Oppositely Charged Donepezil-Loaded Intranasal Liposomes
Source: Pharmaceutics. 2025 Sep 24;17(10):1250. doi: 10.3390/pharmaceutics17101250 (PMC12567290; doi:10.3390/pharmaceutics17101250)
Supplement: Supplementary file 1 [file pharmaceutics-17-01250-s001.zip › pharmaceutics-3840848-supplementary.pdf]

pharmaceutics

MDPI

Supplementary Materials: In Vitro Comparative Study on Oppositely Charged Donepezil-Loaded Intranasal Liposomes

Elika Valehi, Gábor Katona , Dorina Gabriella Dobó and Ildikó Csóka

Table S1. Drug release kinetic models and kinetic parameters of DPZ-loaded liposomes.

Kinetic Model	Kinetic Parameters	PC:CH:DCP 7:2:0.5	PC:CH:DCP 7:2:1	PC:CH:DCP 7:2:1.5	PC:CH:DCP 7:2:2	PC:CH:SA 7:2:0.5	PC:CH:SA 7:2:1	PC:CH:SA 7:2:1.5	PC:CH:SA 7:2:2	DPZ
Zero order	k_0 (μg min^{-1})	0.192	0.310	0.384	0.436	0.429	0.388	0.334	0.224	0.569
	R^2	0.338	0.507	0.591	0.556	0.608	0.828	0.822	0.790	0.341
	$t_{0.5}$ (min)	260.01	161.23	130.21	114.60	116.47	129.00	149.61	223.11	87.81
First order	$k_1 \times 10^{-2}$ (min^{-1})	0.170	0.360	0.54	0.710	0.710	0.550	0.420	0.220	2.460
	R^2	0.655	0.752	0.847	0.844	0.888	0.834	0.808	0.727	0. 946
	$t_{0.5}$ (min)	407.73	192.54	128.361	97.63	97.63	126.03	165.04	315.07	28.18
Higuchi	k_H (μg $min^{-1/2}$)	2.148	1.367	1.125	0.98	1.01	1.111	1.286	1.888	0.738
	R^2	0.916	0.885	0.884	0.899	0.884	0.962	0.9646	0.972	0.910
	$t_{0.5}$ (min)	541.79	1338.42	1973.91	2567.85	2446.38	2025.04	1511.67	701.50	4591.40
Korsmeyer-Peppas	$k_{K\text{-}P}$ (min^{-n})	963.38	108.24	51.19	15.61	61.390	79.360	111.92	272.46	0.5661
	n	1.26	0.86	0.80	0.76	0.79	0.83	0.88	1.00	0.57
	R^2	0.578	0.818	0.871	0,877	0.876	0.862	0.822	0.694	0.903
	$t_{0.5}$ (min)	96.58	407.3	426.22	459.06	773.00	574.35	400.27	184.23	781.45
Hixon-Crowell	$k_{H\text{-}C} \times 10^{-2}$ ($μg^{1/3}$ min^{-1})	0.34	0.60	0.82	1.01	0.990	0.830	0.670	0.400	2.63
	R^2	0.395	0.597	0.725	0.723	0.777	0.869	0.855	0.808	0.932
	$t_{0.5}$ (min)	281.63	159.59	116.76	94.80	96.73	115.37	142.92	239.39	36.41
Best fit		Higuchi	Higuchi	Higuchi	Higuchi	First order	Higuchi	Higuchi	Higuchi	First order

 https://doi.org/10.3390/pharmaceutics17101250
